# Effect of personality traits on driving style: Psychometric adaption of the multidimensional driving style inventory in a Chinese sample

**DOI:** 10.1371/journal.pone.0202126

**Published:** 2018-09-06

**Authors:** Yuchen Wang, Weina Qu, Yan Ge, Xianghong Sun, Kan Zhang

**Affiliations:** 1 CAS Key Laboratory of Behavioral Science, Institute of Psychology, Beijing, China; 2 Department of Psychology, University of Chinese Academy of Sciences, Beijing, China; University of Warsaw, POLAND

## Abstract

Driving style is an important factor in driving psychology, which is useful for effectively evaluating driving behaviors. Exploring driving style in a Chinese sample could help understand the overall situation of Chinese drivers. This study aims to develop a measurement of driving style fit for the Chinese situation and to validate the validity and reliability of this scale. In this study, 296 drivers from China completed the Chinese version of the multidimensional driving style inventory (MDSI), the Big Five Inventory (BFI), and the Driver Behavior Questionnaire (DBQ) as well as several questions about socio-demographic information. After testing the assumed structure by a confirmatory factor analysis, and adjusting the structure, a brief version of Chinese version of MDSI with twelve items categorized into four driving styles, namely, risky style, angry- high-velocity style, careful style and anxious style was revised. The validity and reliability of the scales were acceptable. The results showed that driving styles were closely correlated with self-reported driving behaviors. Specifically, risky style, angry- high-velocity style, and anxious style are all positively associated with dangerous driving behaviors. Meanwhile, careful style was positively associated with positive driving behaviors and negatively correlated with dangerous driving behaviors. Anxious was also found to be negatively associated with fines. For personality, we found a positive relationship between risky, angry- high-velocity, careful and anxious styles and the personality traits that often have negative effects on driving, such as extraversion and neuroticism. Meanwhile, these three styles were negatively correlated with conscientiousness and agreeableness in general. In addition, careful style was positively correlated with conscientiousness and agreeableness. The current study shows convincing evidence for the validity and reliability of the brief MDSI-C and develops a useful tool for identify the driving style of Chinese drivers for future research and relevant departments of road safety.

## Introduction

Every year, more than one million two hundred thousand people are killed by road traffic injuries, which is a leading cause of preventable deaths. In particular, low- and middle-income countries suffer the most road traffic injuries. Ninety percent of deaths in road traffic injuries occurred in low- and middle-income countries, while these countries only own fifty-four percent of the registered vehicles in the world [1]. As a developing country, China also faces the serious problem of road safety. Data from the National Bureau of Statistics of the People’s Republic of China showed that, just in 2015, more than one hundred eighty-seven thousand accidents occurred in China, which caused the deaths of more than fifty-eight thousand people [2]. In addition, China is facing more serious problems of road safety compared with most other countries. According to the WHO, China consistently has the highest estimated number of road traffic fatalities throughout the world in recent years, which reached 261367 in 2015. And the WHO estimated rate of road traffic deaths per 100 000 population of China is 18.8, which is also at a high level compared to most of other countries [1]. Therefore, it is necessary and urgent to pay more attention to road safety problems in China.

Driving style is a driving-relevant variable that comprehensively summarizes behaviors and perceptions while driving and is an important human factor correlated with traffic accidents. As previous research has shown that human factors make up eighty-five percent of the reasons for traffic crashes [3], it is necessary to explore this issue deeply and widely. Elander and co-workers [4] defined driving style in 1993 for the first time as the way an individual habitually drives, including choosing driving speed, obedience to the rules, level of attentiveness and assertiveness. Compared with a single behavior or perception while driving, driving style tends to be a more integrative trait, and it has been confirmed to have a better capacity for predicting unsafe driving behaviors and accident rates of drivers, since drivers with some certain driving styles such as reckless and careless are more likely to perform dangerous driving behavior, even involve in traffic accidents [5–9]. Thus, the exploration of driving style can help us understand more about the causes of certain kinds of driving behavior and outcomes from a more general perspective, since driving style is an index similar to situation trait, and has been proved to be a steady predictor of driving behavior and outcomes [7, 10–11]. It can also help the drivers’ classification, so that drivers with different driving styles could be given personalized driving courses which are more efficient. And the certain groups of people who are more likely to be involved in unsafe driving could be distinguished to avoid working at public traffic positions. These two visions can help improve the traffic situation in China.

### The measurement of driving styles

Though the importance of driving style is widely realized, the conceptualization and measurement of driving style did not reach a consensus for a long time until the development of the multidimensional driving style inventory (MDSI) [7]. Before the development of the MDSI, there were a variety of scales used to measure the behavior and cognition of drivers, such as the Driving Behavior Inventory (DBI) [12,13], Driving Style Questionnaire (DSQ) [14], the Attitudes to Driving Violations (ADVS) [15], Driver Behavior Questionnaire (DBQ) [16], and Driving Vengeance Questionnaire (DVQ) [17]. Since these scales focused on diverse aspects of driving style, the development of the MDSI gave driving style an integrative conceptualization and a multidimensional measuring tool [7]. The original items of the MDSI were mainly adopted from several scales about driving behavior and cognition, such as the DSQ, DBQ and DBI. From this basis, the authors also adjusted the structure and items of the inventory and eventually formed the original version of MDSI.

The original version of MDSI included four general domains with eight specific styles [7,8]. The first domain is reckless and careless, including risky and high-velocity driving styles. This domain assesses deliberate violations of safe driving standards and the sensation-seeking and thrills experienced while driving, including the item *“enjoy the excitement of dangerous driving”*. The second domain is angry and hostile, which includes only the angry style and reflects expressions of rage, irritation, and hostility in a driving context as well as aggressive behavior on the road. *“Swear at other drivers”* is a sample behavior of this domain. The third domain is the anxious domain, composed of anxious, distress-reduction and dissociative driving styles, which refers to alertness and tension feelings along with ineffective engagement in relaxing activities while driving, including feelings such as *“feel nervous while driving”*. The fourth domain is the patient and careful domain, which consists of patient and careful styles and describes driving behaviors that are well-adjusted. *“Plan long journeys in advance”* is a sample behavior belonging to this domain. As the driving styles of the reckless and careless domain, the angry and hostile driving domain and the anxious domain are always associated with unsafe driving behaviors, and previous studies have considered them to be maladaptive driving styles, while the styles of the patient and careful domain, which always correlate with positive driving behaviors, were named adaptive driving styles [7,10].

The MDSI has been used and verified in different cultures around the world, including a Spanish version (MDSI-S) [10] and a Romanian version (MDSI-RO) [11]. The MDSI-S includes six styles. Among those styles, the dissociative, angry, risky, careful, anxious, and distress-reduction styles are ultimately the same as the original version. The patient style was included in the careful style, and some of the items of the high-velocity style were merged into the risky style and the careful style. The MDSI-RO added some items that are fit for the cultural background of Romania. It divided driving style into seven styles, among which the anxious, risky, angry, distress-reduction, and dissociative styles are similar to the original version. A new style named Violation of Rules Contextually Perceived as Irrational Style, was formed mainly by new items. In general, the derived versions of the MDSI all have several differences in the division of the styles of the original version, but the main styles are ultimately blended in all MDSI inventories. Since China is the biggest developing country with the highest population and the second highest car ownership in the world, the traffic condition in China is different from the countries where the MDSI have been developed or revised [18, 19]. As the traffic condition, living environment, and culture background are different around the world, drivers may show different styles when participate in the traffic environment, so it is necessary to explore the driving styles of Chinese drivers systematically. However, to our knowledge, there is no Chinese version of the MDSI that has been validated until now.

### Driving styles and driving behaviors

Driving style has been confirmed to have a stable association with driving behaviors and outcomes. Previous studies have consistently shown that the three maladaptive domains are positively associated with the frequency of reckless driving and the proneness to reckless driving [8]. For example, the reckless and careless domain was positively correlated with the rate of risky events recorded by an in-vehicle data recorder (IVDR) [20]. Drivers of the angry and hostile domain were more likely to have traffic offenses than those of anxious domain [11]. The reckless and careless domain and the angry and hostile domain were found to be positively associated with involvement in car accidents [7] and negatively associated with the assessment of reckless driving [8]. The results of the anxious domain were inconsistent. Some researchers found it was positively correlated with the frequency and proneness of reckless driving [8], but another study showed it was negatively correlated with the rate of risky events recorded by an IVDR [20].

Regarding the adaptive domain, the styles of the patient and careful domain were found to be positively associated with the assessment of reckless driving [8]. It was also found that drivers who scored higher on adaptive styles showed lower frequency of reckless driving, proneness to reckless driving [8], IVDR-recorded rates of risky events [20] and involvement in car accidents [7].

### Driving styles and personality traits

A number of studies have explored the relationship between personality and driving styles. The four driving style domains show some common and specific correlations with different personalities.

The reckless and careless domain is a domain with maladaptive styles correlated with unsafe driving behaviors. On one hand, this manner of driving is positively associated with traits including impulsive sensation-seeking [7,21], aggression-hostility [21], normlessness, discomfort intolerance, driving anger, sensation-seeking and driving thrill-seeking [11]. On the other hand, reckless and careless styles were found to be negatively associated with self-esteem, extraversion [7], agreeableness, conscientiousness [22], and dutifulness [11].

Regarding the angry and hostile driving domain, drivers who possess such driving styles always desire to compete with others on the road and are more likely to have violent behaviors. Positive correlations were found between the angry and hostile driving domain and impulsive sensation-seeking, aggression-hostility [21], normlessness, discomfort intolerance, driving anger, sensation-seeking and driving thrill-seeking [11]. The personalities negatively associated with this manner of driving style were agreeableness, conscientiousness [22] and dutifulness [11], which are widely considered to be adaptive and healthy personalities.

Anxious and dissociative is another maladaptive domain. Drivers with an anxious driving style often have no confidence in their driving abilities and regard other drivers as an additional source of stress [7]. The anxious domain was positively associated with several personalities related to anxiety, including trait anxiety [7], neuroticism [22] and neuroticism-anxiety [21]. People with high scores in anxious styles also score higher on impulsive sensation-seeking [21] as well as driving-relevant traits including discomfort intolerance, driving anger and driving thrill-seeking [11]. The anxious domain was found to be negatively associated with conscientiousness [22] and dutifulness [11].

The patient and careful driving domain is an adaptive and healthy driving style representing higher levels of empathy and care for others, attention to safety, and obedience to the rules. This style was found to be positively associated with self-esteem [7], agreeableness, conscientiousness, openness [22], dutifulness and desire for control [11] and negatively associated with trait anxiety [7], impulsive sensation-seeking, aggression-hostility [21], normlessness, discomfort intolerance, driving anger, sensation-seeking and driving thrill-seeking [11].

### Driving styles and socio-demographic variables

Individual differences also have some impact on the driving style of a driver. Most studies consistently show that with increasing age, the scores of dissociative, anxious, risky and angry, and high-velocity styles, most of which are considered to be maladaptive styles, gradually decrease, and the scores of adaptive styles such as patient and careful styles increase instead [7,11,21,22]. Regarding gender, women are more likely to drive in an anxious or careful driving style, while men score higher in risky and anger styles [8]. Taubman-Ben-Ari and his co-workers also found that driving style is associated with educational background. Positive correlations were found between educational backgrounds and dissociative, anxious, and distress-reduction driving styles [7].

Driving experience is a socio-demographic variable relevant to driving, which mainly consist of the number of driving years and driving mileages. Previous studies have shown that driving years was positively associated with high-velocity driving style and negatively associated with dissociative, anxious and patient styles. Thus, the more experience a driver has, the lower the levels of dissociative, anxious and patient styles and the higher the level of high-velocity style [23,24]. Regarding driving mileages, negative correlations were found between the total and weekly driving mileages and anxious styles [7].

### Aims of the current study

There are three aims of the current study. The first is to develop the Chinese version of multidimensional driving style inventory (MDSI). The reliability, structure and validity of this inventory will be examined so we can determine which types of driving styles are typical among Chinese drivers. The second aim of our research is to explore the relationship between driving styles and personality traits (using the Big Five Inventory, BFI) to determine which types of driving styles are more likely to appear in a person with certain personality traits. Finally, correlations between driving style and socio-demographic variables will be assessed in a Chinese sample.

## Method

### Participants

Participants were recruited online through WeChat (one of the most popular social medias of China, like the Facebook of America), and the data was collected online from September to November in 2016. All the participants need to satisfy the following conditions: (1) at least 18 years old; (2) having a valid driving license; and (3) having at least one year of driving experience. The 336 drivers participating in the current study distributed in several provinces of China, including Beijing, Shandong, Jiangshu, etc. 40 drivers were excluded from analysis because of incompleteness or inconsistencies. The final sample included 296 drivers, aged from 20 to 56 (M = 35.05, SD = 8.57). Men accounted for 60.81% of the sample. In regard to educational background, 10.81% of the participants had an educational level of high school or less, 55.07% had a bachelor’s degree or college education background, and 34.12% had a master’s or doctoral degree. The descriptive statistics for socio-demographic variables are shown in Table 1.

**Table 1:** Descriptive statistics for socio-demographic variables, the BFI, the DBQ, and the MDSI.

Scales	Items	*M*	*SD*	Range (Min-Max)	Cronbach's alpha
Socio-demographic variables					
Age	-	35.05	8.566	20.00–56.00	-
Driving years	-	6.367	5.3643	1.00–30.00	-
Total mileages (KM)	-	6294.70	23279.90	0.00–200000.00	-
Yearly mileages (KM)	-	8968.11	13373.48	0.30–120000.00	-
Weekly mileages (KM)	-	366.81	760.47	0.00–10000.00	-
Accidents	-	1.32	1.49	0.00–8.00	-
Penalty points	-	2.51	3.65	0.00–30.00	-
Fines	-	239.54	340.23	0.00–2200.00	-
The MDSI	12				
Risky	3	2.24	1.02	1.00–5.40	0.801
Angry	3	3.46	0.98	1.00–6.00	0.691
Careful	3	4.78	0.65	2.00–6.00	0.690
Anxious	3	2.08	0.91	1.00–5.33	0.738
The BFI	44				
Extraversion	8	3.16	0.58	1.63–4.88	0.684
Neuroticism	8	2.58	0.66	1.00–4.38	0.751
Openness	10	3.48	0.60	1.70–5.00	0.759
Conscientiousness	9	3.68	0.56	2.00–5.00	0.758
Agreeableness	9	3.85	0.55	1.78–5.00	0.794
The DBQ	41				
Positive behavior	13	4.55	0.54	2.92–5.69	0.674
Aggressive violation	3	2.82	1.17	1.00–6.00	0.830
Ordinary violation	9	2.16	0.77	1.00–5.00	0.826
Error	8	2.00	0.77	1.00–4.75	0.881
Lapse	8	2.31	0.76	1.00–4.75	0.803

Notes: Risky = Risky driving style; Angry = Angry- high-velocity driving style; Careful = Careful driving style; Anxious = Anxious driving style; Regulative = Regulative driving style; Positive = Positive behaviors; Aggressive = Aggressive violations behaviors; Ordinary = Ordinary violations behaviors; Accidents = Accidents in the last three years; Points = Penalty points received in the last year; Fines = Fines received in the last year.

### Measures

#### The multidimensional driving style inventory (MDSI)

The Chinese version of the MDSI (MDSI-C) used in this study was developed based on the original MDSI [7], the Spanish version MDSI-S [10] and the Romania version MDSI-RO[17]. The original author permitted the use of it. The translation process followed the translation/back-translation procedure suggested by Brislin [25] and Regmi et al. [26]. First, three assistant professors in psychology translated the MDSI into Chinese concurrently and independently. After finishing the translations, they had a discussion on their translations and worked out a single draft to make sure its accuracy, fluency and suitability for Chinese driving culture. Second, the instrument was back-translated into English by a professional translator who was proficient in both Chinese and English in order to evaluate the correctness of the translation. Then the three assistant professors and the professional translator discussed the items with ambiguous meanings in a group and modified the items until all members reached an agreement. Finally, three drivers with a driving experience of more than five years were invited to pre-test the translated scale. All the three drivers finished the questionnaire and showed that all the items are understandable. After this procedure, the formal scale was finalized. The final Chinese MDSI included 35 items, some items which are incompatible with the traffic situation of China were excluded for measurement. Some of the items which are incompatible with the traffic situation of China were discarded, such as “Exceed the 50 km/h speed limit in villages on perfectly straight roads with no obstacles limiting my visibility” from the Romanian version, for there is no unifying speed limit in villages of China. In addition, only one of the several items which describe similar situations was left, such as “Running a red light for going along traffic” and “I drive through a traffic light that has just turned red as I was following the car right in front of me”. Only the latter is included in our original inventory. Table 2 showed the descriptive statistics for each items.

**Table 2:** Descriptive statistics for each items, and the factor belonging and ITCs of each items in MDSI, MDSI-S and MDSI-RO.

	Items	*M*	*SD*	MDSI		MDSI-S		MDSI-RO	
				Factor	ITCs	Factor	ITCs	Factor	ITCs
**1**	intend to switch on the windscreen wipers, but switch on the lights instead	2.14	1.219	dissociative	0.638**	dissociative	0.623**	dissociative	0.667**
**9**	forget that my lights are on full beam until flashed by another motorist	2.60	1.245	dissociative	0.612**	dissociative	0.644**	dissociative	0.692**
**17**	nearly hit something due to misjudging my gap in a parking lot	3.07	1.281	dissociative	0.593**	dissociative	0.608**	dissociative	0.632**
**25**	attempt to drive away from traffic lights in third gear (or on the neutral mode in automatic cars)	2.48	1.529	dissociative	0.589**	dissociative	0.572**	dissociative	0.633**
**32**	lost in thoughts or distracted, I fail to notice someone at the pedestrian crossings	2.06	1.179	dissociative	0.710**	dissociative	0.723**	-	-
**34**	I daydream to pass the time while driving^#^	2.32	1.247	dissociative	0.549**	distress-reduction	0.548**	-	-
**3**	**enjoy the excitement of dangerous driving**	2.03	1.391	risky	0.836**	risky	0.796**	risky	0.782**
**11**	**enjoy the sensation of driving on the limit**	2.13	1.395	risky	0.858**	risky	0.830**	risky	0.822**
**19**	**like to take risks while driving**	1.89	1.148	risky	0.801**	risky	0.750**	risky	0.768**
**27**	like the thrill of flirting with death or disaster	1.33	.851	risky	0.661**	**-**	-	-	-
**2**	**feel nervous while driving**	2.29	1.208	anxious	0.759**	anxious	0.703**	anxious	0.782**
**10**	**feel distressed while driving**	2.05	1.128	anxious	0.746**	anxious	0.745**	anxious	0.823**
**18**	**driving makes me feel frustrated**	1.89	1.027	anxious	0.753**	anxious	0.768**	anxious	0.835**
**26**	it worries me when driving in bad weather	4.24	1.216	anxious	0.546**	**-**	-	-	-
**4**	swear at other drivers	2.34	1.324	angry	0.757**	angry	0.757**	-	-
**12**	when someone does something on the road that annoys me, I flash them with the high beam	3.08	1.545	angry	0.804**	**-**	-	-	-
**20**	honk my horn at others as a way of expressing frustrations	2.92	1.529	angry	0.782**	**-**	-	angry high-velocity	0.700**
**5**	**in a traffic jam, I think about ways to get through the traffic faster**^#^	3.95	1.275	high-velocity	0.681**	**-**	-	angry- high-velocity	0.705**
**13**	when in a traffic jam and the lane next to me starts to move, I try to move into that lane as soon as possible^#^	3.15	1.314	high-velocity	0.719**	**-**	-	angry- high-velocity	0.700**
**21**	**when a traffic light turns green and the car in front of me doesn’t get going immediately, I try to urge the driver to move on**^#^	3.86	1.323	high-velocity	0.711**	**-**	-	angry- high-velocity	0.766**
**28**	purposely tailgate other drivers^#^	2.78	1.367	high-velocity	0.550**	risky	0.510**	-	-
**33**	**get impatient during rush hours**^#^	3.44	1.389	high-velocity	0.690**	anxious	0.566**	angry- high-velocity	0.719**
**35**	drive through traffic lights that have just turned red^#^	1.81	1.014	high-velocity	0.490**	angry	0.617**	-	-
**6**	use muscle relaxation techniques while driving	4.12	1.236	distress-reduction	0.738**	**-**	-	distress-reduction	0.781**
**14**	while driving, I try to relax myself	4.61	1.049	distress-reduction	0.731**	**-**	-	distress-reduction	0.748**
**22**	do relaxing activities while driving	3.92	1.303	distress-reduction	0.677**	distress-reduction	0.763**	distress-reduction	0.702**
**29**	mediate while driving	4.60	.896	distress-reduction	0.576**	distress-reduction	0.458**	-	-
**7**	**at an intersection where I have to give right-of-way to oncoming traffic, I wait patiently for cross-traffic to pass**^#^	4.99	1.003	patient	0.681**	careful	0.645**	patient and careful	0.721**
**8**	**drive cautiously**	5.30	0.832	patient	0.578**	**-**	-	patient and careful	0.668**
**15**	**base my behavior on the motto “better safe than sorry”** ^#^	5.25	0.981	patient	0.581**	careful	0.724**	patient and careful	0.660**
**16**	always ready to react to unexpected maneuvers by other drivers	4.69	1.061	patient	0.662**	careful	0.718**	patient and careful	0.592**
**23**	when a traffic light turns green and the car in front of me doesn’t get going, I just wait for a while until it moves^#^	3.68	1.336	patient	0.613**	angry	-0.726**	patient and careful	0.528**
**24**	distracted or preoccupied, and suddenly realize the vehicle ahead has slowed down, and have to slam on the breaks to avoid a collision^#^	3.12	1.209	patient	0.670****	dissociative	-0.145*	-	-
**30**	plan long journeys in advance^#^	5.08	1.077	patient	0.565**	careful	0.604**	-	-
**31**	get a thrill out of breaking the law	1.29	0.714	patient	0.556**	**-**	-	-	-

Notes

^#^ the item belong to different styles in different version of MDSI

the items adopted in the final inventory are bolded

* *p* < 0.05

** *p* < 0.01.

The participants were required to evaluate their thoughts, feelings and behaviors while driving on a 6-point Likert scale, ranging from *not at all* (1) to *very much* (6). After confirming and adjusting the structure of the inventory, the Cronbach's alpha of the final four subscales were higher than 0.60.

#### The Big Five Inventory (BFI)

Personality was measured by the Chinese version of Big Five Inventory (BFI) [27] which was developed from the BFI by John et al. [28]. This inventory consisted of forty-four items divided into five styles, including extraversion, neuroticism, openness, conscientiousness and agreeableness. Participants were asked to rate their agreement on a 5-point scale, ranging from *strongly disagree* (1) to *strongly agree* (5). In the current study, the Cronbach's alpha of the five subscales were all higher than 0.75 except the extraversion dimension, which also had an acceptable value of0.68.

#### The Driver Behavior Questionnaire (DBQ)

The DBQ used here included aberrant behaviors and positive behaviors. Aberrant driving behavior was measured by the 28-item Chinese version of the DBQ [29], which was developed on the basis of the extended version of the DBQ [30,31]. Positive driving behavior was measured by the 13-item positive driving behavior scale developed by Özkan and Lajunen [32]. This scale was also verified in a Chinese sample [33]. Therefore, in this study, the full version of the DBQ included 41 items with 5 dimensions: Ordinary Violations, Aggressive Violations, Errors, Lapses, and Positive Behavior. Each item described a type of behavioral expression. Participants were required to choose the frequency that best matched their behaviors, from *never* (1) to *always* (6). Higher scores indicated a higher frequency of the given behavior. The Cronbach's alpha of the five subscales were all above 0.80 in the current sample except Positive Behavior, which had a Cronbach's alpha that was also acceptable (0.674).

### Procedure

After recruiting the participants through Wechat, the data was collected online by a professional online assessment system called Sojump, which is widely used in China. The commit time, completion time, IP address and city are recorded by the system to make sure the returned questionnaires are reliable.

The participants were required to finish a series of questionnaires including the MDSI, BFI and DBQ anonymously. After finishing all the scales, the participants provided their demographic information including age, gender, driving years, accidents in the last three years, penalty points and fines received in the last year. Each participant needed approximately 15 minutes to complete the questionnaire and received CNY 15 (approximately USD 2.3) for their participation. This study was approved by the Institutional Review Board of the Institute of Psychology, Chinese Academy of Sciences.

## Results

### Internal structure

Confirmatory factor analysis (CFA) was used to test the internal structure of the Chinese MDSI Via Amos 24.0. The following indices were adopted to evaluate the goodness-of-fit of the models. A root mean squared error of approximation (RMSEA) less than 0.08 was considered acceptable [34]. During the iterative revision process, the Joreskog Sorbom goodness-of-fit index (GFI), Bentler’ s comparative fit index (CFI), and the Tucker–Lewis index (TLI) was evaluated and all the three index should be higher than 0.90 in a satisfied fitted model [35]. Finally, the Akaike information criterion (AIC) value was evaluated and lower AIC values were considered better.

First, the eight-factor structure of the original version of MDSI, the six-factor structure of the Spanish version of MDSI and the six-factor structure of the Romania version of MDSI (as the items of “Violation of rules contextually perceived as irrational” factor of the MDSI-RO were all absent in the initial selection, only six factors were left in the structure of MDSI-RO) were tested. The results revealed a poor fit of these two models (Table 3).

**Table 3:** Model fit of the different versions of MDSI.

Scales	RMSEA	*χ2/df*	GFI	CFI	TLI	AIC	CAIC
MDSI	0.076	2.725	0.784	0.707	0.672	1645.692	2105.347
MDSI-S	0.077	2.769	0.840	0.757	0.717	782.319	1077.811
MDSI-RO	0.083	3.018	0.826	0.749	0.713	991.016	1305.270
MDSI (3 items)	0.093	3.558	0.826	0.743	0.684	948.984	1305.452
MDSI-S (3 items)	0.072	2.525	0.902	0.857	0.817	405.017	644.225
MDSI-RO (3 items)	0.075	2.675	0.894	0.856	0.817	422.961	662.169
MDSI-RO (3 items, 4 factors)	0.067	2.337	0.943	0.934	0.909	172.163	312.874

Notes: MDSI = the modified original version of MDSI with 35 seleted items; MDSI-S = the modified Spanish version of MDSI with 30 selected items; MDSI-RO = the modified Romania version of MDSI with 27 selected items; MDSI (3 items) = the modified original version of MDSI with three items per style; MDSI-S (3 items) = the modified Spanish version of MDSI with three items per style; MDSI-RO (3 items) = the modified Romania version of MDSI with three items per style; MDSI-RO (3 items, 4 factors) = the modified Romania version of MDSI with four factors consist of 3 items respectively.

Then, in order to improve the model fit and make the scale to be more convenient to use in research and practical situations, we decided to validate a brief version of the MDSI-C (brief MDSI-C). First, according to the previous study, the three items with the highest item-total correlation coefficients (ITCs) for each factor were selected to make a short version scale [36–37]. The model fit of the modified original, Spanish and Romania version MDSI were tested again. The model fits of these three versions were better than the original ones. And the brief Spanish version and Romania version were better than the brief original version. Because the GFI, CFI of brief MDSI-S and MDSI-RO were all higher than 0.85, and the RMSEA of brief MDSI-S and MDSI-RO were lower than 0.08, while the GFI, CFI, TLI and RMSEA of brief version of original MDSI were all unacceptable. Second, we checked the reliabilities of each factors in the modified MDSI-S and MDSI-RO. In the brief MDSI-S, the reliabilities of distress reduction (0.104) angry (0.486) and distress reduction (0.484) were lower than 0.60, which are unacceptable. As for brief MDSI-RO, the reliabilities of dissociative style (0.489) and distress reduction (0.586) are unacceptable. So these factors were abandoned in the final version. In order to evaluate driving styles of Chinese drivers in a more diversified way, we think the structure brief MDSI-RO is more suitable for Chinese version of MDSI for there are more kinds of styles remained in brief MDSI-RO. Third, the model fit of the new four-factor structure was tested again and the model fit was acceptable (Table 3). Thus, the brief MDSI-C with four factors was used for further analyses.

### Descriptive statistics and reliability

The mean (*M*), standard deviation (*SD*), range (Min-Max) and the index of Cronbach's alpha for socio-demographic variables, four subscales of the MDSI, five subscales of the BFI, and five subscales of the DBQ are shown in Table 1. All the scales showed acceptable internal consistency reliability [38].

### Correlation analysis and validity

The correlations between each variable are shown in Table 4. The content validity was examined by the relationship between the brief MDSI-C and DBQ subscales. The criterion validity was tested by correlations between brief MDSI-C and self-reported accidents, penalty points and fines. Additionally, the relationship between the MDSI and the BFI was also measured to explore the relationship between driving styles and personality.

**Table 4:** Correlations among the MDSI, the BFI, the DBQ, accidents, penalty points and fines.

	1	2	3	4	5	6	7	8	9	10	11	12	13	14	15	16	17	18	19
1 Age	1																		
2 Sex	0.009	1																	
3 Driving years	0.589**	-0.176**	1																
4 Extraversion	-0.109	0.148*	-0.038	1															
5 Neuroticism	-0.053	0.183**	-0.090	-0.311**	1														
6 Openness	-0.137*	-0.036	-0.019	0.294**	-0.283**	1													
7 Conscientiousness	0.077	-0.042	0.051	0.219**	-0.547**	0.340**	1												
8 Agreeableness	0.052	0.048	-0.003	0.082	-0.484**	0.242**	0.453**	1											
9 Risky	-0.067	-0.150**	-0.003	0.046	0.152**	0.068	-0.265**	-0.285**	1										
10 Angry	0.000	-0.096	0.058	0.116*	0.232**	0.068	-0.205**	-0.309**	0.316**	1									
11 Careful	-0.010	0.150**	-0.088	0.072	-0.146*	0.087	0.289**	0.289**	-0.312**	-0.115*	1								
12 Anxious	-0.108	0.077	-0.132*	-0.131*	0.344**	-0.203**	-0.312**	-0.307**	0.210**	0.077	-0.288**	1							
13 Positive	0.042	0.037	0.060	-0.021	-0.051	0.152**	0.190**	0.232**	-0.171**	-0.148*	0.318**	-0.156**	1						
14 Aggressive	-0.009	-0.110	0.057	0.001	0.256**	-0.004	-0.252**	-0.401**	0.377**	0.599**	-0.218**	0.133*	-0.245**	1					
15 Ordinary	0.057	-0.079	0.079	-0.030	0.195**	-0.054	-0.292**	-0.377**	0.541**	0.392**	-0.349**	0.256**	-0.192**	0.525**	1				
16 Error	-0.056	0.025	-0.027	0.053	0.234**	-0.114*	-0.266**	-0.303**	0.359**	0.219**	-0.374**	0.493**	-0.253**	0.295**	0.664**	1			
17 Lapse	0.010	0.192**	-0.067	-0.030	0.313**	-0.170**	-0.394**	-0.306**	0.317**	0.189**	-0.241**	0.504**	-0.179**	0.286**	0.625**	0.706**	1		
18 Accidents	-0.099	-0.046	-0.059	0.029	-0.017	0.011	-0.058	-0.102	0.143*	0.071	-0.089	-0.042	-0.014	0.058	0.091	0.082	0.037	1	
19 Points	0.020	-0.013	0.063	0.149*	-0.034	0.013	-0.063	-0.048	0.211**	0.115*	-0.216**	-0.020	-0.046	0.105	0.254**	0.101	0.162**	0.244**	1
20 Fines	-0.047	-0.075	-0.002	-0.025	0.095	0.058	-0.116*	-0.080	0.202**	0.165**	-0.115*	-0.078	-0.174**	0.142*	0.205**	0.042	0.086	0.195**	0.547**

Notes: Risky = Risky driving style; Angry = Angry- high-velocity driving style; Careful = Careful driving style; Anxious = Anxious driving style; Positive = Positive behaviors; Aggressive = Aggressive violations behaviors; Ordinary = Ordinary violations behaviors; Accidents = Accidents in the last three years; Points = Penalty points received in the last year; Fines = Fines received in the last year.

* p < 0.05.

** p < 0.01.

There are significant correlations between each two of the four styles of the brief MDSI-C. The careful style is negatively associated with the other three styles, and the risky, dissociative and anxious styles are positively associated with each other. The careful style is considered as adaptive driving styles, and the other three styles are considered as maladaptive driving styles.

The results of the relationships between the brief MDSI-C and the DBQ show that all three maladaptive styles are negatively correlated with the positive behaviors dimension and positively correlated with other dimensions of the DBQ. In contrast, careful driving style was positively correlated with the positive behaviors dimension and negatively correlated with other dimensions of the DBQ. In addition, the risky style was positively associated with accidents, penalty points and fines. No significant correlations were found between other three styles of brief MDSI-C and accidents, penalty points, fines.

Regarding the personality traits, all the maladaptive driving styles are positively associated with neuroticism and negatively associated with conscientiousness and agreeableness. In addition, dissociative and anxious driving style is negatively correlated with the openness dimension, anxious driving style is negatively associated with extraversion. For the careful driving style, a negative correlation was found with neuroticism, and positive correlations were found with all other dimensions of the BFI.

In regard to socio-demographic variables, self-reported males reported higher risky scores. Meanwhile, the driving years is negatively associated with anxious style. No significant associations were found between the other two driving styles and socio-demographic variables.

### Multivariate multiple regression analysis

To explore the relationship among personality traits, brief MDSI-C, driving behaviors and driving outcomes, a multivariate multiple regression analysis was used via Amos 24.0. The assumed model consists of four hierarchies of variables. In the first hierarchy, the BFI personalities including extraversion, neuroticism, openness, conscientiousness and agreeableness were added. In the second hierarchy, the four driving styles risky, dissociative, careful and anxious were included. In the third hierarchy, the five dimensions of DBQ including positive behavior, ordinary behavior, aggressive behavior, error and lapse were added. And the driving outcomes including penalty points and fines were added as the dependent variables. The variables in one hierarchy were assumed correlated with each other. The variables in the former hierarchy were assumed to have effect on the variables in all latter hierarchies. The paths between two variables which were not significant were deleted. The final model showed very good model fit: RMSEA = 0.036, *χ*^*2*^/*df* = 1.392, GFI = 0.962, CFI = 0.983, TLI = 0.970, AIC = 230.648, CAIC = 549.592. The index of each significant path of this model were showed in Tables 5–7.

**Table 5:** The multivariate multiple regression index (*β*) of BFI personalities on MDSI.

		Risky	Angry- high-velocity	Careful	Anxious
BFI Personalities	Extraversion	-	-	-	0.142*
	Neuroticism	-	0.189**	-	0.267***
	Openness	0.197***	0.143*	-	-
	Conscientiousness	-0.196**	-	0.176**	-
	Agreeableness	-0.245***	-0.262***	0.210***	-0.176**

Notes: *β =* standardized regression weights

*p < 0.05

**p < 0.01

***p < 0.001

“-” means the regression weight is not significant (*p* > 0.05) and the relationship is considered inexistent.

**Table 6:** The multivariate multiple regression index (*β*) of BFI personalities, MDSI on driving behaviors.

		Positive	Aggressive	Ordinary	Error	Lapse
BFI Personalities	Extraversion	-	-	-	0.102**	-
	Neuroticism	-	-	-	-	-
	Openness	0.127*	-	-	-	-
	Conscientiousness	-	-	-	-	-0.194***
	Agreeableness	-	-0.198***	-0.126**	-	-
Brief MDSI-C	Risky	-	0.162***	0.423***	0.222***	0.198**
	Angry- high-velocity	-	0.480***	0.161***		
	Careful	0.297***	-	-0.147***	-0.200***	-
	Anxious	-	-	-	0.361***	0.369***

Notes: *β =* standardized regression weights

*p < 0.05

**p < 0.01

***p < 0.001; “-” means the regression weight is not significant (*p* > 0.05) and the relationship is considered inexistent.

**Table 7:** The multivariate multiple regression index (*β*) of BFI personalities, MDSI, DBQ on driving outcomes.

		Penalty Points	Fines
BFI Personalities	Extraversion	0.185***	-
	Neuroticism	-	-
	Openness	-	-
	Conscientiousness	-	-
	Agreeableness	-	-
Brief MDSI-C	Risky	-	-
	Angry- high-velocity	-	-
	Careful	-0.145**	-
	Anxious	-	-0.106*
DBQ	Positive	-	-0.177***
	Aggressive	-	-
	Ordinary	0.201***	0.192***
	Error	-	-
	Lapse	-	-

Notes: *β =* standardized regression weights

*p < 0.05

**p < 0.01

***p < 0.001

“-” means the regression weight is not significant (*p* > 0.05) and the relationship is considered inexistent

The results show that personality traits have effect on driving styles. More specifically, risky style was positively predicted by openness, and negatively predicted by conscientiousness and agreeableness. Angry and high-velocity style was positively associated by neuroticism and inversely associated by openness and agreeableness. Careful driving style was related to conscientiousness and agreeableness traits positively. Anxious style was positively predicted by extraversion and neuroticism, and negatively associated by agreeableness.

In addition, controlling for personalities, the driving style of brief MDSI-C also had some effect on driving behaviors and driving outcomes. As for driving behavior, the results show that the risky style had positive effect on all the driving behaviors of DBQ except positive behavior. The angry and high-velocity style had positive effect on ordinary and aggressive behavior. The careful driving style could positively influence on positive driving behavior, and inversely influence on ordinary and error behavior. The anxious style could positively predict error and lapse behavior. The relationship between driving styles and driving outcomes are also explored. The results show that anxious style could predict fines inversely, and careful style have negative effect on penalty points. No other effects are shown between driving styles and driving outcomes.

## Discussion

Based on a sample of Chinese drivers, the current study developed a brief Chinese version of the MDSI, which consists of four driving styles of Chinese people. The brief MDSI-C showed sufficient reliability and validity, and our results highlighted the relationship between the MDSI and personalities.

First, the brief MDSI-C has good reliability and a stable structure. The CFA results showed that the brief MDSI-C includes four styles, risky style, angry- high-velocity style, careful style, and anxious style. All four styles had acceptable internal consistency. The four styles structure is mainly derived from the MDSI-RO. The three driving styles violation of rules contextually perceived as irrational, dissociative and distress reduction are unfit and delected in the brief MDSI-C, while the other driving styles are same as the MDSI-RO. The absence of the violation of rules contextually perceived as irrational factor is because the rules mentioned in the items are not fit for the Chinese situation. The remove of dissociative and distress reduction styles may due to the special Chinese driving condition. As a country with the second highest car ownership, China has a very busy traffic condition [18, 19]. Such traffic condition demand the drivers to keep alert all the time on the road. So the dissociative and distress reduction styles are incompatible with the traffic situation of China. In summary, the brief MDSI-C styles are different with the original version to some extent but have basically covered the original styles. Internal connections were also found between the four driving styles. In general, the three maladaptive styles have positive connections between each other and are inversely connected with the only adaptive style. These results agree with former studies [10].

The validity of the MDSI-C was confirmed by the relationship between the brief MDSI-C and driving behaviors and outcomes. As for the results between driving style and driving behavior, in general, the current research found that the maladaptive driving styles are positively associated with the negative driving behaviors but negatively correlated with positive driving behaviors. The adaptive driving style is positively associated with the positive driving behavior and inversely associated with the negative behaviors. According to these results, people with careful driving styles are more likely to perform positively while driving, and people with the other three maladaptive driving styles tend to drive in maladaptive ways. These results are almost consistent with other studies such as the research by Taubman-Ben-Ari et al. [7] and Taubman-Ben-Ari et al. [20]. The results indicate that driving style is different with specific driving behavior, which is more general and may be causes of driving behaviors. Different driving styles could lead to different driving behaviors, while one certain driving behavior may be influenced by several different styles. In addition, careful style was found to be a negative prediction of penalty points, which means that the adaptive driving style leads to less offense of driving. The negative relationship between anxious style and fines were also found. Although anxious is not a maladaptive style in our usual impression, anxious people also tend to perform cautiously while driving, and this may cause less fines. The result indicates that there may be also some positive respect on maladaptive styles. The contribution of driving style is shown as careful and anxious styles still have significant effects on driving outcome while controlling the effects of driving behavior. To our surprise, the other two driving styles did not show effects on the driving outcomes, while previous research found that other driving style are also associated with accidents, offenses and other driving outcomes [7, 11]. This may because of the disadvantage of self-reported method. Some participants may conceal their real situation of driving outcomes because of social desirability effect, which suggest the future research to use the data from official agencies or real observation. Therefore, we can see that driving style is a consistently good predictor of driving behaviors, and driving outcomes to some extent, which means that the newly revised brief MDSI-C has good validity.

As for personality traits, the results shown that maladaptive driving styles are generally positively associated with extraversion, neuroticism and openness and negatively related to conscientiousness and agreeableness. In contrast, the adaptive driving style is positively related to conscientiousness and agreeableness. The results imply that personality has a significant association with driving styles. Driving styles tend to be stable traits of one’s driving characteristics, linking personality and driving behavior. The current results mainly agree with the research of Taubman-Ben-Ari and Yehiel [22]. However, we found that openness is positively associated with risky and angry- high-velocity driving styles, but other researchers have found a positive correlation between openness and careful driving style [22]. The correlation between openness and sensation-seeking supports our results to some degree. Several studies have consistently found that openness is positively associated with sensation-seeking in several conditions [39–40]. Since sensation-seeking is a trait stably related to risky and angry- high-velocity driving [41–42], it makes sense that people with higher openness may perform more risky and angry- high-velocity driving behaviors.

The current study also found several relationships between socio-demographic variables and driving styles by the correlation method. The risky style is negatively associated with self-reported respondent sex, and careful style is positively associated with self-reported respondent sex, which is essentially consistent with previous studies [7,23]. The gender differences in driving style are similar to the differences in driving behaviors, which can partly explain the different driving tendencies of men and women, such as men being more likely to be involved in speeding violations [43]. Anxious style was found to be negatively associated with driving years, which means that more experiences in driving can reduce the anxiety of driving. The result is also in line with the previous studies [7,23]. The limitations of the current study should be noted. First, the data of the current study were mainly collected by self-report, so social desirability [44], the misremembering of the actual feeling, thought and behavior may have some impact on the the results. Although researchers have demonstrated that self-reported data are as useful as archival driving data [45] and data from field studies [46–47], future studies should integrate self-report measures with other methods such as field observations and simulated driving. In addition, adopting the self-reported behavior as the criterion may limit the reliability of the test’s validity. In order to avoid the subjectivity and shared method variance of the research, the future research had better use the data from official agencies or real observation (such as the experiments on real-life driving or car-simulator driving) as the criterion for testing the validity of MDSI-C. Second, we used a convenience sampling method to recruit volunteers to participate in the current study, and the sample size is still not enough for a more defensible inventory revision. The sampling method of our research may lead to a sampling bias to some extent. Therefore, future research would enlarge the sample and to choose participants from all areas and from all conditions using a stratified random sampling method. Third, the reliability should be further tested in the future research by a test-retest exercise, since only one test has been done in the current study.

In conclusion, the current study examined the reliability and validity of the MDSI-C, which can be a useful tool for research about human factors in the field of road safety in China. In practice, while driving style can stably predict dangerous driving behaviors, the MDSI-C can help the relevant departments identify the causes of unsafe driving behaviors. The relationships already examined between personality, socio-demographic variables and driving style in a Chinese sample can provide important support for the classifying of drivers, so that we can identify the drivers with certain maladaptive driving styles who are more likely to be involved in risky driving behaviors and traffic accidents, and the further measures should be taken. Moreover, individualized driving education can be developed. We can also get a better understanding about the causes of certain kinds of driving behavior and outcomes from a more general perspective. Since China is still a country with growing traffic and a high rate of traffic accidents, the revision of the MDSI-C can contribute to the improvement of road safety in China both theoretically and practically.

## Supporting information

S1 Questionnaire The MDSI questionnaire (English and Chinese version). (DOCX)

S1 Original Data The original data used in the current study. (XLSX)
