# Supplementary material for: Effect of personality traits on driving style: Psychometric adaption of the multidimensional driving style inventory in a Chinese sample
Source: PLoS One. 2018 Sep 6;13(9):e0202126. doi: 10.1371/journal.pone.0202126 (PMC6126823; doi:10.1371/journal.pone.0202126)
Supplement: S1 Questionnaire — (DOCX) [file pone.0202126.s001.docx]

**Instruction:** please read the following items carefully, and consider how well do the items reflect your feeling, thought and behavior while driving. Then choose the appropriate option.

| 1 | intend to switch on the windscreen wipers, but switch on the lights instead | 1 2 3 4 5 6 |
| --- | --- | --- |
| 2 | feel nervous while driving | **1 2 3 4 5 6** |
| 3 | enjoy the excitement of dangerous driving | **1 2 3 4 5 6** |
| 4 | swear at other drivers | **1 2 3 4 5 6** |
| 5 | in a traffic jam, I think about ways to get through the traffic faster | **1 2 3 4 5 6** |
| 6 | use muscle relaxation techniques while driving | **1 2 3 4 5 6** |
| 7 | at an intersection where I have to give right-of-way to oncoming traffic, I wait patiently for cross-traffic to pass | **1 2 3 4 5 6** |
| 8 | drive cautiously | **1 2 3 4 5 6** |
| 9 | forget that my lights are on full beam until flashed by another motorist | **1 2 3 4 5 6** |
| 10 | feel distressed while driving | **1 2 3 4 5 6** |
| 11 | enjoy the sensation of driving on the limit | **1 2 3 4 5 6** |
| 12 | when someone does something on the road that annoys me, I flash them with the high beam | **1 2 3 4 5 6** |
| 13 | when in a traffic jam and the lane next to me starts to move, I try to move into that lane as soon as possible | **1 2 3 4 5 6** |
| 14 | while driving, I try to relax myself | **1 2 3 4 5 6** |
| 15 | base my behavior on the motto “better safe than sorry” | **1 2 3 4 5 6** |
| 16 | always ready to react to unexpected maneuvers by other drivers | **1 2 3 4 5 6** |
| 17 | nearly hit something due to misjudging my gap in a parking lot | **1 2 3 4 5 6** |
| 18 | driving makes me feel frustrated | **1 2 3 4 5 6** |
| 19 | like to take risks while driving | **1 2 3 4 5 6** |
| 20 | honk my horn at others as a way of expressing frustrations | **1 2 3 4 5 6** |
| 21 | when a traffic light turns green and the car in front of me doesn’t get going immediately, I try to urge the driver to move on | **1 2 3 4 5 6** |
| 22 | do relaxing activities while driving | **1 2 3 4 5 6** |
| 23 | when a traffic light turns green and the car in front of me doesn’t get going, I just wait for a while until it moves | **1 2 3 4 5 6** |
| 24 | distracted or preoccupied, and suddenly realize the vehicle ahead has slowed down, and have to slam on the breaks to avoid a collision [−] | **1 2 3 4 5 6** |
| 25 | attempt to drive away from traffic lights in third gear (or on the neutral mode in automatic cars) | **1 2 3 4 5 6** |
| 26 | it worries me when driving in bad weather | **1 2 3 4 5 6** |
| 27 | like the thrill of flirting with death or disaster | **1 2 3 4 5 6** |
| 28 | purposely tailgate other drivers | **1 2 3 4 5 6** |
| 29 | mediate while driving | **1 2 3 4 5 6** |
| 30 | plan long journeys in advance | **1 2 3 4 5 6** |
| 31 | get a thrill out of breaking the law [−] | **1 2 3 4 5 6** |
| 32 | lost in thoughts or distracted, I fail to notice someone at the pedestrian crossings | **1 2 3 4 5 6** |
| 33 | get impatient during rush hours | **1 2 3 4 5 6** |
| 34 | I daydream to pass the time while driving | **1 2 3 4 5 6** |
| 35 | drive through traffic lights that have just turned red | **1 2 3 4 5 6** |

**指导语：**请认真阅读每一道题目，并思考它们多大程度上反映了您在驾驶过程中的感受、想法及行为，选择适合的描述。

| 1 | 想打开雨刷，却打开了灯 | 1 2 3 4 5 6 |
| --- | --- | --- |
| 2 | 驾驶时感觉紧张 | **1 2 3 4 5 6** |
| 3 | 享受危险驾驶带来的的快感（刺激感） | **1 2 3 4 5 6** |
| 4 | 骂其他司机 | **1 2 3 4 5 6** |
| 5 | 交通拥堵时，我会想方设法尽快驶离 | **1 2 3 4 5 6** |
| 6 | 在驾驶时，放松肌肉 | **1 2 3 4 5 6** |
| 7 | 在十字路口，当我必须给前方的车让路时，我会耐心等待 | **1 2 3 4 5 6** |
| 8 | 谨慎驾驶 | **1 2 3 4 5 6** |
| 9 | 忘了自己开了大灯，直到被其他驾驶者闪灯提醒 | **1 2 3 4 5 6** |
| 10 | 驾驶时感觉痛苦 | **1 2 3 4 5 6** |
| 11 | 享受极限驾驶的感觉 | **1 2 3 4 5 6** |
| 12 | 当其他车惹恼我时，我会用大灯晃它 | **1 2 3 4 5 6** |
| 13 | 交通拥堵时，如果旁边的车道开始移动，我会尽快插入 | **1 2 3 4 5 6** |
| 14 | 在驾驶时，试着让自己放轻松 | **1 2 3 4 5 6** |
| 15 | 我在开车时以“确保安全，不留遗憾”为原则。 | **1 2 3 4 5 6** |
| 16 | 随时做好准备应对其他车辆的突发行为 | **1 2 3 4 5 6** |
| 17 | 在停车时，因为错判了间距，差点撞到东西 | **1 2 3 4 5 6** |
| 18 | 驾驶使我沮丧 | **1 2 3 4 5 6** |
| 19 | 驾驶时喜欢冒险 | **1 2 3 4 5 6** |
| 20 | 对其他车猛按喇叭以表达不满 | **1 2 3 4 5 6** |
| 21 | 绿灯亮了而前边的车还没有动，我会催促它 | **1 2 3 4 5 6** |
| 22 | 在驾驶时，做一些能让人放松的事 | **1 2 3 4 5 6** |
| 23 | 当绿灯亮了，但我前边的车没有移动时，我会耐心等待 | **1 2 3 4 5 6** |
| 24 | 由于分心，驾驶时突然意识到前车减速而不得不急刹车（-） | **1 2 3 4 5 6** |
| 25 | 在红灯变绿灯时，用空档状态启步。 | **1 2 3 4 5 6** |
| 26 | 恶劣天气下的驾驶，让我担忧 | **1 2 3 4 5 6** |
| 27 | 喜欢面临死亡或灾难时恐惧的快感 | **1 2 3 4 5 6** |
| 28 | 紧紧跟着前边的车辆 | **1 2 3 4 5 6** |
| 29 | 在驾驶时调节自己 | **1 2 3 4 5 6** |
| 30 | 我会提前规划长途旅行。 | **1 2 3 4 5 6** |
| 31 | 因为违章驾驶而感到兴奋（-） | **1 2 3 4 5 6** |
| 32 | 因为发呆或分心，没有没有注意到人行横道上的行人。 | **1 2 3 4 5 6** |
| 33 | 在高峰期感到不耐烦 | **1 2 3 4 5 6** |
| 34 | 在驾驶时胡思乱想打发时间。 | **1 2 3 4 5 6** |
| 35 | 红灯刚亮时，闯过去 | **1 2 3 4 5 6** |
